# Approach to Fatigue in Primary Care: A Practical Diagnostic Framework for General Practitioners

**DOI:** 10.7759/cureus.108764

**Published:** 2026-05-13

**Authors:** Omar Elbaroumi

**Affiliations:** 1 General Practice, NHS Independent Practice, Colchester, GBR

**Keywords:** chronic fatigue, diagnosis, family medicine, fatigue, long covid, nice guidelines, primary care

## Abstract

Fatigue is one of the most common presenting complaints in primary care and poses a significant diagnostic challenge due to its multifactorial aetiology. While the majority of cases are benign and self-limiting, fatigue may also represent an early manifestation of serious underlying pathology. This review distinguishes between acute fatigue, typically transient and associated with intercurrent illness or lifestyle factors, and chronic fatigue, defined as fatigue persisting for six or more weeks, which is more likely to be multifactorial in origin.

This narrative review aims to provide a practical and structured diagnostic framework for general practitioners to evaluate and manage fatigue effectively in the primary care setting.

A narrative review of the literature was conducted using PubMed and Google Scholar. Searches were limited to articles published in English from 2010 onwards. Search terms included "fatigue," "primary care," "chronic fatigue," "myalgic encephalomyelitis," "post-viral fatigue," "sleep disorders," and "functional somatic syndromes." Seminal references predating 2010 were retained where no suitable replacement was available. This review did not employ a formal systematic search strategy, and no risk-of-bias assessment was performed, consistent with the narrative review format.

Fatigue arises from a wide range of physical, psychological, and lifestyle-related causes, best understood through a three-tier classification: primary/idiopathic, secondary, and psychosocial. A systematic approach incorporating thorough history-taking, focused clinical examination, and judicious use of investigations is essential. Identification of red flag symptoms is critical to exclude serious conditions, including malignancy and chronic infections.

A structured, patient-centred approach enables general practitioners to manage fatigue effectively while minimising unnecessary investigations and ensuring timely identification of serious disease.

## Introduction and background

Fatigue is a frequently encountered symptom in primary care, accounting for a substantial proportion of general practitioner consultations [1,2]. It represents a significant burden on healthcare systems and is associated with reduced quality of life, impaired functional capacity, and increased healthcare utilisation [1-3]. Patients often describe fatigue as a persistent sense of tiredness, reduced energy, or exhaustion that is not relieved by rest [4].

In many cases, fatigue is associated with benign or self-limiting conditions such as poor sleep, stress, and physical deconditioning [5]. However, it may also represent the early presentation of significant medical conditions, including endocrine disorders, chronic infections, autoimmune disease, or malignancies [6]. Distinguishing between these possibilities is a key challenge for general practitioners, particularly in the context of time-limited consultations.

An important early distinction is between acute fatigue and chronic fatigue. Acute fatigue is typically transient, associated with intercurrent illness, infection, or short-term lifestyle disruption, and is often self-limiting. Chronic fatigue, defined as fatigue persisting for six or more weeks, is more likely to be multifactorial in origin and may involve an interplay of physical, psychological, and social factors [3,7]. This review focuses primarily on the assessment and management of fatigue in primary care, with particular attention to chronic or persistent presentations.

Mental health conditions, particularly depression and anxiety, are frequently associated with fatigue and may complicate both diagnosis and management [8,9]. Modern lifestyle factors, including sedentary behaviour, sleep disruption, and substance use, have contributed to the increasing prevalence of fatigue in the general population.

Primary care clinicians must balance the need to exclude serious pathology with the risk of over-investigation. Excessive testing may lead to incidental findings, increased patient anxiety, and unnecessary healthcare costs. Clinical guidance, including that provided by the National Institute for Health and Care Excellence (NICE), emphasises a structured approach incorporating clinical assessment, identification of red flags, and targeted investigations [4,5,10-12].

This review aims to provide a practical and structured framework, presented as a narrative clinical review, for the assessment and management of fatigue in primary care, with a focus on supporting safe, efficient, and evidence-based clinical decision-making.

Methods

A narrative review of the literature was conducted using PubMed and Google Scholar. Searches were limited to articles published in English from 2010 onwards. Search terms included: "fatigue," "primary care," "chronic fatigue," "myalgic encephalomyelitis," "chronic fatigue syndrome," "post-viral fatigue," "sleep disorders," and "functional somatic syndromes." Approximately 80 articles were retrieved across all searches, of which approximately 40 were selected for inclusion based on relevance to the assessment and management of fatigue in primary care.

Inclusion criteria were peer-reviewed articles or clinical guidelines relevant to fatigue in primary care, in the English language and published from 2010 onwards. Seminal references predating 2010 were retained where no suitable equivalent was identified. Exclusion criteria included non-English language publications, case reports, and studies focused exclusively on secondary or tertiary care settings. No formal systematic search strategy, PRISMA-style screening process, or risk-of-bias assessment was performed, consistent with the narrative review format. Article selection was performed by the lead author.

## Review

Causes of fatigue

Fatigue can arise from a wide range of causes and is best understood using a structured, three-tier classification: primary/idiopathic, secondary, and psychosocial (Table 1). In many patients, fatigue is not attributable to a single cause but reflects an interaction between multiple contributing factors. Recognising whether fatigue is acute or chronic is an essential first step, as it guides the differential diagnosis and the approach to investigation and management [3].

**Table 1:** Common causes of fatigue in primary care. This table summarises the major categories of fatigue encountered in primary care using a three-tier classification: primary/idiopathic, secondary, and psychosocial. Psychosocial causes are listed separately, given their clinical importance and distinct management approach, though they may overlap with secondary causes in some classification systems. These categories frequently overlap, reflecting the multifactorial nature of fatigue in clinical practice [3,5,6,8,9,11].

Category	Examples	Reference
Primary/Idiopathic	Myalgic encephalomyelitis/Chronic fatigue syndrome, fibromyalgia, idiopathic chronic fatigue	[5,9]
Secondary	Haematological: anaemia, leukaemia; Endocrine: hypothyroidism, hyperthyroidism, diabetes mellitus, Addison's disease; Cardiovascular: heart failure, arrhythmia; Respiratory: chronic obstructive pulmonary disease (COPD), obstructive sleep apnoea; Infectious: tuberculosis, HIV, Epstein-Barr virus; Autoimmune: systemic lupus erythematosus (SLE), rheumatoid arthritis; Neurological: multiple sclerosis, post- cerebrovascular accident (CVA) fatigue; Malignancy: any systemic cancer; Medication-induced: antihypertensives, sedatives, antihistamines, antidepressants	[3,6]
Psychosocial	Depression, anxiety, burnout, somatisation disorder, occupational stress, substance use and addiction	[8,11]

Primary (idiopathic) causes include myalgic encephalomyelitis/chronic fatigue syndrome (ME/CFS) and fibromyalgia. These conditions are characterised by persistent, debilitating fatigue that is not explained by another medical diagnosis and is often accompanied by cognitive difficulties, pain, and sleep disturbance [5,9]. ME/CFS, in particular, is associated with post-exertional malaise, a worsening of symptoms following physical or mental exertion, which has important implications for management and distinguishes it from other causes of fatigue. A multidisciplinary approach involving medical, psychological, and rehabilitative support is typically required [5,9].

Secondary causes encompass a broad range of organ system pathologies. Haematological causes, including anaemia, are among the most common reversible causes of fatigue in primary care [3]. Endocrine disorders, particularly hypothyroidism and diabetes mellitus, may impair metabolic function and energy production. Cardiovascular and respiratory conditions such as heart failure and obstructive sleep apnoea can significantly impair oxygenation and sleep quality, contributing to persistent fatigue. Circadian rhythm disorders may further exacerbate symptoms by disrupting normal sleep-wake cycles [12]. Chronic infections, including tuberculosis, HIV, and Epstein-Barr virus, are important considerations, particularly in patients with systemic symptoms. Autoimmune conditions such as systemic lupus erythematosus and rheumatoid arthritis frequently present with fatigue as an early or prominent feature [3]. Neurological conditions, including multiple sclerosis and post-stroke fatigue, should also be considered, particularly when accompanied by focal neurological symptoms. Malignancy-related fatigue is an important consideration in patients with constitutional symptoms. Finally, medication-induced fatigue should not be overlooked; commonly prescribed drugs, including antihypertensives, antidepressants, antihistamines, and sedatives, can contribute significantly to symptoms [3].

Psychosocial causes are highly prevalent and frequently under-recognised. Depression, anxiety, burnout, and somatisation disorders may present predominantly with physical symptoms such as fatigue, rather than overt psychological complaints, making diagnosis challenging [11]. The relationship between fatigue and mental health is complex and bidirectional; fatigue may both result from and contribute to psychological distress [11]. Substance use, including alcohol and recreational drugs, is an important and often overlooked contributor to chronic fatigue and should be actively inquired about in all patients [3]. Occupational stress, caregiving responsibilities, and significant life events may also contribute substantially to symptom development.

Recognising the multifactorial and often overlapping nature of these categories is essential in guiding a holistic, patient-centred approach to diagnosis and management

Initial assessment

A structured initial assessment is the cornerstone of evaluating fatigue in primary care. This involves a systematic approach encompassing comprehensive history-taking, focused physical examination, and the judicious use of validated screening tools, providing the foundation upon which further investigation and management decisions are based.

Clinical assessment

A structured clinical assessment is essential in the evaluation of fatigue and should begin with a comprehensive history. Distinguishing between acute and chronic fatigue is particularly important, as acute fatigue is more commonly associated with infections or transient lifestyle factors, whereas chronic fatigue is often multifactorial [3].

Key aspects of the history include the duration and onset of symptoms, severity, and the degree of functional impairment, including the impact on daily activities, work, and social functioning. Sleep patterns and sleep quality should be explored in detail, including symptoms suggestive of sleep disorders such as insomnia or obstructive sleep apnoea, as sleep disturbance is both a cause and consequence of fatigue [12].

Psychological symptoms, including low mood, anxiety, and stress, should be assessed systematically. Validated screening tools such as the Patient Health Questionnaire-9 (PHQ-9) and Generalized Anxiety Disorder-7 (GAD-7) are useful in identifying underlying depression and anxiety [11,13]. The STOP-BANG (Snoring, Tiredness, Observed apnoea, high blood Pressure, Body mass index, Age, Neck circumference, and Gender) questionnaire may be used to screen for obstructive sleep apnoea in patients with relevant risk factors [12].

A thorough medication review is essential, as many commonly prescribed drugs, including sedatives, antidepressants, antihypertensives, and antihistamines, may contribute to fatigue [3]. Substance use, including alcohol and recreational drugs, should also be assessed. Social and occupational history, including work patterns, shift work, and lifestyle factors, may provide important diagnostic clues.

Physical examination should include assessment of vital signs, general inspection for features such as pallor, weight loss, or cachexia, and focused examination of the cardiovascular, respiratory, endocrine, and neurological systems [3]. Particular attention should be paid to signs that may indicate systemic disease, such as lymphadenopathy, thyroid enlargement, or neurological deficits.

Red flags

Having established a structured clinical assessment framework, it is essential to recognise specific features that may indicate serious underlying pathology requiring urgent action. Although the majority of cases encountered in primary care are benign and self-limiting, the presence of specific clinical features may indicate serious underlying pathology requiring urgent evaluation (Table 2) [4].

**Table 2:** Red flag features in fatigue. This table outlines key red flag symptoms that may indicate serious underlying pathology in patients presenting with fatigue. The presence of these features should prompt urgent investigation or referral in accordance with national guidance [4]. STOP-BANG: Snoring, Tiredness, Observed apnoea, high blood Pressure, Body mass index, Age, Neck circumference, and Gender

Red Flag Symptom	Possible Underlying Cause	Clinical Action	Reference
Unintentional weight loss	Malignancy, chronic infection	Urgent investigation (bloods ± imaging)	[4]
Persistent or unexplained fever	Infection (e.g. tuberculosis), inflammatory disease	Further evaluation and blood tests	[4]
Night sweats	Tuberculosis, lymphoma	Consider urgent referral	[4]
Lymphadenopathy	Malignancy, chronic infection	Examination ± imaging/biopsy	[4]
Unexplained bleeding or bruising	Haematological disorders (e.g. leukaemia)	Urgent blood tests	[4]
Unexplained anaemia	Malignancy, gastrointestinal blood loss, nutritional deficiency	Urgent bloods and investigation of the cause	[3,4]
Chest pain or palpitations	Cardiac arrhythmia, heart failure	ECG, cardiology review	[3]
Dyspnoea on exertion	Heart failure, respiratory disease, severe anaemia	Clinical examination, ECG, echocardiogram	[3]
Severe or refractory sleep disturbance	Obstructive sleep apnoea, psychiatric disorder	STOP-BANG screening, sleep study referral	[12]
Neurological deficits (focal weakness, sensory changes)	Multiple sclerosis, post- cerebrovascular accident (CVA) fatigue	Urgent referral and imaging	[3]

Red flag symptoms should be assessed systematically during the clinical history and examination. Unintentional weight loss, particularly when significant or progressive, may suggest malignancy, chronic infection, or systemic disease. Persistent or unexplained fever and night sweats are concerning for infections such as tuberculosis or endocarditis, as well as haematological malignancies, including lymphoma [4]. Lymphadenopathy, especially when persistent, generalised, or associated with systemic symptoms, warrants further investigation. Unexplained anaemia, chest pain, palpitations, or dyspnoea on exertion should prompt cardiac and respiratory evaluation. Severe or refractory sleep disturbance may indicate obstructive sleep apnoea and should prompt STOP-BANG screening and referral for sleep studies where appropriate [12].

The presence of multiple red flag symptoms, rapid symptom progression, or significant functional decline increases the likelihood of serious disease and should lower the threshold for urgent referral and investigation. In primary care, effective safety-netting is essential; patients without immediate red flags should be advised to return promptly if new symptoms develop or existing symptoms worsen [4].

Investigations

When to Investigate

The role of investigations in the assessment of fatigue is to identify underlying pathology while avoiding unnecessary testing. A structured and targeted approach is essential, as indiscriminate investigation may lead to incidental findings, increased patient anxiety, and unnecessary healthcare utilisation (Table 3) [14].

**Table 3:** Investigation approach for fatigue in primary care. This table outlines first-line investigations applicable to all patients and second-line investigations guided by clinical findings and history. The selection of further investigations should be directed by examination findings and the presence or absence of red flag features. In patients with red flag symptoms, urgent referral and imaging should be arranged in accordance with national guidance [4]. Over-investigation in low-risk patients should be avoided [14]. PHQ-9: Patient Health Questionnaire-9; GAD-7: Generalized Anxiety Disorder-7; STOP-BANG: Snoring, Tiredness, Observed apnoea, high blood Pressure, Body mass index, Age, Neck circumference, and Gender

Investigation	Indication	What It Identifies	Reference
First-line: All patients			
Full blood count (FBC)	All patients	Anaemia, haematological malignancy, infection	[3,14]
Thyroid function tests (TFTs)	All patients	Hypothyroidism, hyperthyroidism	[3,14]
Fasting glucose/HbA1c	All patients	Diabetes mellitus	[3,14]
Renal function (urea and electrolytes)	All patients	Chronic kidney disease, electrolyte imbalance	[3,14]
Liver function tests (LFTs)	All patients	Hepatic disease, alcohol-related pathology	[3,14]
C-reactive protein/Erythrocyte sedimentation rate	All patients	Infection, inflammatory disease, malignancy	[3,14]
Second-line: Guided by clinical findings			
Iron studies (ferritin, serum iron, total iron-binding capacity)	Suspected iron deficiency; abnormal FBC	Iron deficiency anaemia	[3,14]
Vitamin B12 and folate	Neurological symptoms, macrocytic anaemia, dietary risk	B12/Folate deficiency	[3]
Coeliac screen (tTG-IgA)	GI symptoms, unexplained anaemia, weight loss	Coeliac disease	[3]
HIV/Epstein-Barr virus/Cytomegalovirus serology	Risk factors, systemic symptoms, lymphadenopathy	Chronic infection	[3,6]
Antinuclear antibody/Rheumatoid factor	Suspected autoimmune disease, joint symptoms	Systemic lupus erythematosus , rheumatoid arthritis	[3]
Morning cortisol	Suspected adrenal insufficiency	Addison's disease	[3]
Chest X-ray	Respiratory symptoms, suspected malignancy, tuberculosis risk	Pulmonary pathology, lymphadenopathy	[4]
Abdominal ultrasound	Hepatomegaly, splenomegaly, pelvic symptoms	Organ-specific pathology, malignancy	[4]
Sleep study/STOP-BANG	Suspected obstructive sleep apnoea, refractory fatigue	Sleep-disordered breathing	[12]
PHQ-9/GAD-7	Suspected depression or anxiety	Depressive disorder, anxiety disorder	[11,13]

In patients without red flag features and with a low clinical suspicion of serious disease, a conservative approach to investigation is often appropriate. Over-investigation may lead to false-positive results, unnecessary follow-up testing, and increased healthcare costs. In such cases, a period of watchful waiting, combined with appropriate safety-netting, can be a valuable strategy [14]. Patients should be advised to return for reassessment if symptoms persist, worsen, or if new symptoms develop.

Further investigations should be guided by the clinical history and examination findings. For example, iron studies may be indicated in patients with suspected iron deficiency, while vitamin B12 and folate levels may be appropriate in those with neurological symptoms or dietary risk factors. Coeliac screening should be considered in patients with gastrointestinal symptoms or unexplained anaemia. Imaging studies, such as chest radiography or abdominal ultrasound, may be warranted where there is suspicion of malignancy or organ-specific pathology.

When to Refer

In the presence of red flag symptoms, urgent investigation and referral are required to exclude serious conditions such as malignancy or chronic infection, in accordance with national guidance [4]. Referral to appropriate specialities should be guided by the clinical findings; for example, haematology referral for suspected haematological malignancy, respiratory referral for suspected obstructive sleep apnoea or pulmonary pathology, and rheumatology referral for suspected autoimmune disease

Management

Management of fatigue requires a holistic and patient-centred approach, taking into account the multifactorial nature of the symptom and the individual patient context (Table 4). In many cases, effective management involves addressing a combination of physical, psychological, and lifestyle-related factors rather than a single underlying cause.

**Table 4:** Management framework for fatigue in primary care. This table summarises a holistic, patient-centred approach to the management of fatigue. Lifestyle recommendations, including graded activity, should be applied selectively. Graded exercise is contraindicated in ME/CFS due to the risk of post-exertional malaise and should be used cautiously in patients with active malignancy or significant systemic disease [5,9]. Management should be guided by clinical assessment and supported by appropriate investigation and safety-netting strategies [3,4,11,14].

Component	Approach	Considerations	Reference
Treat the underlying cause	Address identified medical or psychiatric conditions	Targets reversible pathology: anaemia, thyroid disease, depression	[3,14]
Psychological support	Cognitive behavioural therapy (CBT), behavioural activation, and pharmacotherapy for depression/anxiety	CBT is evidence-based for chronic fatigue of any cause	[11]
Lifestyle modification	Sleep hygiene, graded activity (where appropriate), balanced diet, alcohol reduction	Graded exercise is contraindicated in myalgic encephalomyelitis/chronic fatigue syndrome (ME/CFS); tailor to the underlying cause	[3,5]
Substance use	Address alcohol misuse and recreational drug use	Both can perpetuate fatigue and impair sleep quality	[3]
Patient education	Set realistic expectations; explain the multifactorial nature	Improves adherence and reduces frustration	[5]
Follow-up and safety-netting	Regular review; advise return if symptoms worsen or new features develop	Detects evolving pathology; reduces risk of missed diagnoses	[4]

Where an underlying medical cause is identified, treatment should be directed accordingly. For example, anaemia should be treated with appropriate iron supplementation or further investigation of its cause, while endocrine disorders such as hypothyroidism require targeted medical therapy.

Psychological factors should be actively addressed, as depression, anxiety, and stress are common contributors to fatigue. These conditions should be managed using a combination of pharmacological and non-pharmacological approaches. Cognitive behavioural therapy (CBT) is an evidence-based intervention for chronic fatigue of any cause, not solely for depression or anxiety, and should be considered in all appropriate patients [11]. Early recognition and intervention are important, as untreated psychological conditions may perpetuate fatigue and impair recovery.

Lifestyle modification plays a central role in management and should be discussed with all patients. Establishing consistent sleep routines and improving sleep hygiene are particularly important. In patients with suspected obstructive sleep apnoea or circadian rhythm disorders, referral for sleep studies should be considered [12]. Patients should be encouraged to engage in regular physical activity where appropriate. However, activity management must be carefully individualised according to the underlying cause. Graded activity may be beneficial in patients with deconditioning, depression, or lifestyle-related fatigue. Graded exercise is contraindicated in ME/CFS, where it may precipitate or worsen post-exertional malaise, and should be used with caution in patients with active malignancy or significant systemic disease [5,9].

Substance use, including alcohol misuse and recreational drug use, should be actively assessed and addressed as part of the management plan. Both alcohol and recreational substances can directly impair sleep quality, disrupt circadian rhythms, and exacerbate fatigue, and their contribution is frequently underestimated in clinical practice [3]. Brief intervention and onward referral to appropriate support services should be considered where relevant.

Shared decision-making is particularly important in primary care. Engaging patients in discussions about their symptoms, potential contributing factors, and management options can enhance patient satisfaction and promote adherence to treatment plans.

Follow-Up and Safety-Netting

Follow-up and safety-netting are essential components of management. Regular review allows for monitoring of symptom progression, reassessment for emerging pathology, and adjustment of management plans as required. Patients should be advised to seek further medical attention if symptoms persist, worsen, or if new red flag features develop [4].

Discussion

Fatigue remains one of the most challenging symptoms encountered in primary care due to its non-specific nature and broad differential diagnosis [1-3,8,14]. While most cases are benign, the potential for fatigue to represent serious disease necessitates a structured and cautious approach. A key challenge lies in balancing over-investigation and under-diagnosis, as excessive testing may lead to incidental findings and increased patient anxiety, whereas insufficient evaluation risks delayed diagnosis of serious illness [14].

The multifactorial nature of fatigue further complicates management, as physical, psychological, and social factors frequently coexist. This supports the need for a biopsychosocial model of care [15,16], although applying this approach in practice can be challenging, particularly when patients expect a purely biomedical explanation for their symptoms. Effective communication and patient-centred care are therefore essential.

ME/CFS remains an important diagnostic consideration in patients with persistent fatigue and functional impairment [17]. Clinicians should be aware of the contraindication of graded exercise therapy in ME/CFS, given the risk of post-exertional malaise. In recent years, increasing attention has focused on post-viral fatigue, particularly following COVID-19 infection, which has become an increasingly relevant issue in primary care [18,19]. The long-term implications of post-viral fatigue remain uncertain and represent an important area for future research.

Continuity of care is a major strength of general practice, allowing for longitudinal assessment and early detection of evolving pathology. In the context of increasing patient demand and limited consultation time, general practitioners must rely on structured assessment frameworks to support safe and effective clinical decision-making.

This review has several limitations. As a narrative clinical review, no formal systematic search strategy, PRISMA-style screening, or risk-of-bias assessment was performed. The literature search, while broad, may not have captured all relevant evidence. Some references predating 2010 have been retained where no suitable contemporary equivalent was identified. Readers should interpret findings in this context and refer to current national guidance for definitive clinical recommendations.

## Conclusions

Fatigue is a common yet complex presentation in primary care that requires a structured and patient-centred approach. Given its broad differential diagnosis, clinicians must balance the need to identify serious underlying pathology with the risk of over-investigation. A systematic assessment incorporating thorough history-taking, focused examination, and targeted investigations is essential in guiding appropriate clinical decision-making.

A three-tier classification of causes, primary/idiopathic, secondary, and psychosocial, provides a practical framework to guide the differential diagnosis. A holistic management strategy that addresses physical, psychological, lifestyle, and substance use factors can improve patient outcomes. Clinicians should be mindful that graded activity recommendations must be individualised, and that ME/CFS requires a specifically tailored approach due to the risk of post-exertional malaise. Continuity of care and effective safety-netting remain critical in ensuring timely reassessment and detection of evolving pathology.
